# Hypoxia-preconditioned mesenchymal stem cells ameliorate ischemia/reperfusion-induced lung injury

**DOI:** 10.1371/journal.pone.0187637

**Published:** 2017-11-08

**Authors:** Yung-Yang Liu, Chi-Huei Chiang, Shih-Chieh Hung, Chih-Feng Chian, Chen-Liang Tsai, Wei-Chih Chen, Haibo Zhang

**Affiliations:** 1 Chest Department, Taipei Veterans General Hospital, Taipei, Taiwan; 2 Department of Medicine, School of Medicine, National Yang-Ming University, Taipei, Taiwan; 3 Institute of Clinical Medicine, School of Medicine, National Yang-Ming University, Taipei, Taiwan; 4 Department of Medicine, Tri-Service General Hospital, National Defense Medical Center, Taipei, Taiwan; 5 Integrative Stem Cell Center, Chinese Medical University Hospital, Graduate Institute of Clinical Medical Science, China Medical University, Taichung, Taiwan; 6 Graduate Institute of Clinical Medical Science, China Medical University, Taichung, Taiwan; 7 Department of Orthopaedics & Traumatology, Taipei Veterans General Hospital, Taipei, Taiwan; 8 Therapeutical and Research Center of Musculoskeletal Tumor, Taipei Veterans General Hospital, Taipei, Taiwan; 9 Division of Pulmonary and Critical Care Medicine, Internal Medicine Department, Tri-Service General Hospital, National Defense Medical Center, Taipei, Taiwan; 10 Department of Physiology, and Interdepartmental Division of Critical Care Medicine, University of Toronto, Toronto, Ontario, Canada; University of Cincinnati College of Medicine, UNITED STATES

## Abstract

**Background:**

Hypoxia preconditioning has been proven to be an effective method to enhance the therapeutic action of mesenchymal stem cells (MSCs). However, the beneficial effects of hypoxic MSCs in ischemia/reperfusion (I/R) lung injury have yet to be investigated. In this study, we hypothesized that the administration of hypoxic MSCs would have a positive therapeutic impact on I/R lung injury at molecular, cellular, and functional levels.

**Methods:**

I/R lung injury was induced in isolated and perfused rat lungs. Hypoxic MSCs were administered in perfusate at a low (2.5×10^5^ cells) and high (1×10^6^ cells) dose. Rats ventilated with a low tidal volume of 6 ml/kg served as controls. Hemodynamics, lung injury indices, inflammatory responses and activation of apoptotic pathways were determined.

**Results:**

I/R induced permeability pulmonary edema with capillary leakage and increased levels of reactive oxygen species (ROS), pro-inflammatory cytokines, adhesion molecules, cytosolic cytochrome C, and activated MAPK, NF-κB, and apoptotic pathways. The administration of a low dose of hypoxic MSCs effectively attenuated I/R pathologic lung injury score by inhibiting inflammatory responses associated with the generation of ROS and anti-apoptosis effect, however this effect was not observed with a high dose of hypoxic MSCs. Mechanistically, a low dose of hypoxic MSCs down-regulated P38 MAPK and NF-κB signaling but upregulated glutathione, prostaglandin E2, IL-10, mitochondrial cytochrome C and Bcl-2. MSCs infused at a low dose migrated into interstitial and alveolar spaces and bronchial trees, while MSCs infused at a high dose aggregated in the microcirculation and induced pulmonary embolism.

**Conclusions:**

Hypoxic MSCs can quickly migrate into extravascular lung tissue and adhere to other inflammatory or structure cells and attenuate I/R lung injury through anti-oxidant, anti-inflammatory and anti-apoptotic mechanisms. However, the dose of MSCs needs to be optimized to prevent pulmonary embolism and thrombosis.

## Introduction

Ischemia/reperfusion (I/R) injury is a common clinical problem encountered in lung transplantation, resuscitation from shock and cardiac surgery. I/R injury following organ transplantation can result in primary graft dysfunction, and it is the leading cause of morbidity and mortality after transplantation [1]. Although the molecular events that occur during I/R lung injury are complex, we previously reported two major presentations of I/R lung injury in the early phase: permeability pulmonary edema and acute inflammation [2]. Previous studies have reported that microvascular injuries are evoked by activated endothelial cells and oxidative cell damage, and that a burst of oxidative stress plays an important role in initiating I/R lung injury, which is then followed by inflammation and apoptosis [1–11].

With regards to the molecular mechanisms of I/R lung injury, it has been proposed that I/R can activate cellular transduction [1] resulting in the generation of reactive oxygen species (ROS), nuclear factor-kappa B (NF-κB) translocation, production of inflammatory cytokines [2–6] and the upregulation of cell surface co-stimulatory molecules [7–10]. This sequence is followed by more inflammatory cells being recruited into the interstitium and alveoli. In addition, mitochondrial Ca^2+^ overload and increased ROS can induce the opening of mitochondrial permeability transition pores and the release of cytochrome C from the mitochondria into cytosol, which further compromises cellular energy metabolism and leads to rupture of the plasma membrane and cell death [11].

The administration of mesenchymal stem cells (MSCs) has been shown to repair lung epithelial cells [12–15] and improve pulmonary functions in animal models of endotoxin-induced acute lung injury [16] and in patients with advanced chronic lung allograft dysfunction [17]. The lung protective effects of MSCs may be related to their property of modulating angiogenesis [18] and anti-inflammatory effects [19]. Most studies examining the effects of MSCs have been conducted in animal models of bleomycin and endotoxin-induced lung injury [16, 20–22]. However, the therapeutic potential of MSCs remains largely unknown in I/R lung injury. Furthermore, no prior study has investigated the effect of hypoxic MSCs on I/R lung injury in the early phase.

We previously demonstrated that MSCs expand under hypoxic conditions and display decreased replicative senescence and an increased rate of proliferation and differentiation potential [23]. Moreover, hypoxic MSCs have also been shown to have increased abilities to migrate and engraft after transplantation compared to MSCs [24]. In this study, we hypothesized that the administration of hypoxic MSCs would have a beneficial impact on pulmonary I/R injury via anti-oxidant and anti-inflammatory mechanisms. We demonstrated that hypoxic MSCs can ameliorate I/R lung injury in the acute phase in a rat model, but that a large dose of hypoxic MSCs may induce pulmonary embolism.

## Materials and methods

This study was approved by the institutional board for animal care and use of Taipei Veterans General Hospital (TVGH), Taiwan. All experimental animals were cared for in accordance with the Guide for the Care and Use of Laboratory Animals by the National Institutes of Health of United States. Sixty male Sprague-Dawley (SD) rats weighing 250–350 grams were obtained from the BioLASCO Taiwan Co., Ltd. Rats were housed in ventilated cages (265×420×180 mm) at room temperature and humidity of 50–70% controlled by air conditioning, and maintained with 12 hours of light and 12 hours of darkness at the TVGH Laboratory Animal Center. Rats were fed with autoclaved Reverse Osmosis purified water and Laboratory Autoclavable Rodent Diet 5010. A detailed protocol is described in the online supporting information (S1 File).

### Isolation and expansion of rat MSCs under hypoxic conditions

To isolate SD rats MSCs, bone marrow mononuclear cells were seeded at a density of 10^4^ cells/cm^2^ and cultured under hypoxic (1% O_2_) conditions. At 24 h, non-adherent cells were removed by changes of medium and irrigation of the culture. At 72 hours, some adherent cells were observed (S1A Fig). At 8–9 days, the cells typically formed aggregates with the center reaching confluence (S1B Fig). The cells were then harvested and subcultured at low density (100 cells/cm^2^), where cells always reached confluence 9 days later under hypoxic conditions, when subculture was performed again. The fold increase in cell number with each passage (9-day interval) was only slightly reduced at late passages (S1C Fig); and the population doubling time (PDT) only slightly increased at late passages (S1D Fig). The hypoxic cells could be expanded without loss of proliferation up to 7 to 8 passages. Actually, the cells in hypoxic culture were still tiny and had spindle-shaped morphology. These findings suggested low-density culture combined with hypoxic condition increases proliferation capacity and expansion efficiency in rat MSCs.

### Characterizations of rat MSCs expanded under hypoxic conditions

We assessed the surface marker profiles of MSCs expanded under hypoxic conditions (S2 Fig). These cells were negative for hematopoietic or endothelial markers such as CD11b, CD31 and CD45, but positive for CD29, CD44, CD90, CD106, suggesting these cells possessed putative markers of MSCs. One of the properties of MSCs is to differentiate into osteoblasts, adipocytes and chondrocytes. Therefore, hypoxic MSCs at passage 3 were induced to undergo differentiation into osteoblasts, adipocytes and chondrocytes (S3 Fig). For osteogenic differentiation, Alizarin Red S staining revealed hypoxic MSCs achieved osteogenic differentiation 21 days after induction (S3 Fig). For adipogenic differentiation, Oil Red O staining revealed hypoxic MSCs succeeded in adipogenic differentiation 21 days after induction (S3 Fig). For chondrogenic differentiation, the pellet cultures stained by Alcian Blue and immunohistochemistry revealed that hypoxic MSCs differentiated into chondrocytes and expressed chondrogenic proteins 21 days after induction (S3 Fig). These results suggested rat MSCs expanded under hypoxic conditions possess the normal properties of MSCs.

### Isolated and perfused lung model

The *in situ* isolated-perfused lung model has been previously described [2–3]. Briefly, male SD rats were anesthetized with intraperitoneal injection of sodium pentobarbital during the study period. A tracheotomy was performed and mechanical ventilation was applied (Rodent ventilator Model 683, Harvard Apparatus, South Natick, MA, USA) at a tidal volume of 6 mL/kg and positive end-expiratory pressure (PEEP) of 2 cmH_2_O. The inspired gas used contained 5% CO2 and 95% air. After a sternotomy, heparin (1 unit/g) was injected into the right ventricle through which pulmonary artery was catheterized. The left atrium was catheterized at the apex of the heart. The pulmonary venous outflow was diverted into a reservoir. To prevent flow back into the ventricles an additional ligation was performed above the atrio-ventricular junction. The lungs were perfused with 10 mL blood mixed with 20 mL 0.9% normal saline (Minipulse 2; Gilson Medical Electronic, Middleton, WI, USA) at a constant flow at 30 μl/min/g body weight. Pulmonary arterial pressure (P_pa_), pulmonary venous pressure (P_pv_), pH in circulating perfusate and peak airway pressure were monitored. The rat weight was determined to reflect lung weight in the *in situ* system and recorded the lung weight gain (LWG) continuously. Pulmonary arterial resistance (*R*_a_) and venous resistance (*R*_v_) were calculated using the following equations: *R*_a_ = (P_pa_—P_pc_) / Q, and *R*_v_ = (P_pc_—P_pv_) / Q, where Q is perfusate flow. At the end of the study period, all experimental animals were sacrificed by exsanguination under anesthesia and all efforts were taken to minimize animal suffering.

### Study groups

The *in situ* isolated-perfused lungs were ventilated at a tidal volume of 6 ml/kg for 2 hours in all rats, the following four groups were designed and each group contain 7 rats (N = 7): (1) Control group: same operation of isolated reperfused rat lung with mechanical ventilation without receiving hypoxic MSCs and I/R challenge; (2) I/R group: ischemia one hour then reperfusion one hour and receiving vehicle control solution (same volume of normal saline as MSCs infusion); (3) I/R+MSCL group: I/R receiving low-dose (2.5×10^5^) hypoxic MSCs in saline solution; and (4) I/R+MSCH group: I/R receiving high-dose (1×10^6^) hypoxic MSCs in saline solution. Ischemia was introduced by stopping ventilation and perfusion for 60 min in the isolated lung preparation followed by reperfusion for 60 min at room temperature. Hypoxic MSCs were administered in the circulating perfusate into pulmonary artery 10 minutes prior to the onset of ischemia.

After complete animal experiment, the left lung was lavaged and the right upper lung was excised for wet/dry ratio assessment and the right lower lung was snap frozen and used for other measurements.

### Measurements

Description of the measurements including inflammatory responses, signaling pathways, apoptosis, and etc. is provided in details in the online supplementary materials. Vascular permeability was evaluated by determining pulmonary capillary filtration coefficient (K_*fc*_) as previously described [9–10].

### Oxidative stress, myeloperoxidase and inflammatory cytokines

The concentration of myeloperoxidase (MPO), an index of neutrophil sequestration was measured in lung tissue [2–3]. The levels of protein carbonyl and thiobarbituric acid reactive substances (TBARS) [2–3] and H_2_O_2_ were measured in bronchoalveolar lavage fluid (BALF). The content of glutathione (GSH) was measured in lung tissue.

The concentrations of cytokines including interleukin-1β (IL-1β), tumor necrosis factor-α (TNF-α), macrophage inflammatory protein 2 (MIP-2), interleukin-10 (IL-10) and prostaglandin E2 were measured in BALF using commercial ELISA kits specific for rats.

### Adhesion molecule, mitogen-activated protein kinases, nuclear factor kappa B, mitochondria and cytochrome C

The expression of intercellular adhesion molecule (ICAM-1) and vascular cell adhesion protein 1 (VCAM-1) was determined in lung tissue by using appropriate antibodies. The phosphorylation of extracellular signal-regulated kinases (ERK), Jun N-terminal kinases (JNK), P38 mitogen-activated protein kinases (MAPK) was analyzed by using specific antibodies in lung tissue. The level of NF-κB was measured in nuclear protein of lung tissue. Mitochondria was isolated from the lung tissue, and the concentration of cytochrome C in mitochondria and cytoplasm was measured.

### Apoptosis

TUNEL stain was performed for assessment of apoptosis in lung tissue. Index of apoptotic pathways including caspase-3 and B-cell lymphoma 2 (Bcl-2) were evaluated in lung tissue by Western blot analysis.

### MSC tracking assay

In additional experiments, MSCs were incubated with 10 μM thymidine analog BrdU for 18 h, prior to being administered. Immunohistochemistry of BrdU-MSCs in lung tissue was conducted by using monoclonal antibody against BrdU (Gene Tex, San Antonio, Texas, USA) followed by biotinylated secondary antibody (Vector Laboratories, Burlingame, CA, USA).

### Protocol of pulmonary embolism measurement

Pulmonary embolism (PE) is a blockage of the artery of the lung by a substance that has travelled from elsewhere in the body through the bloodstream [25]. We developed a scoring method to measure the amount of pulmonary embolism, as follows. Pulmonary embolism was positive vessel obstruction by red cell and other cells. The total number of positive obstructive vessels was counted in 3 slides of hematoxylin/eosin stain in each rat lung tissues. The pulmonary emboli scores were analyzed by a pathologist who was blinded to the experimental groups.

### Histology

Lung injury score was evaluated by a pathologist who was blinded to the study groups [10].

### Statistical analysis

Systat10.0 (Systat Software Inc., San Jose, CA, USA) was used for statistical analysis. Comparisons among groups were analyzed using ANOVA followed by Dunnett's method of post-hoc testing. Comparison between baseline and post-I/R values within group was conducted using Student's paired *t*-test. Values are expressed as mean ± SD. P < 0.05 was considered as statistically significant.

## Results

### Hemodynamics

The I/R, I/R+MSCL and I/R+MSCH groups all induced a higher pulmonary arterial pressure as compared to the control group (Table 1). In addition, the I/R+MSCH group had a greater increase in pulmonary arterial and capillary pressure compared to the other groups (Table 1).

**Table 1:** Hemodynamics.

Group	N	*P*_*Pa*_	*P*_*Pv*_	*P*_*Pc*_	*R*_*a*_	*R*_*v*_
**Before injury**						
Control	7	13.83±1.96	0.42±0.64	6.56±0.92	0.16±0.03	0.12±0.03
I/R	7	13.79±1.91	0.42±0.64	6.41±0.75	0.16±0.02	0.12±0.02
I/R + MSCL	7	13.86±2.01	0.43±0.72	6.58±0.98	0.16±0.05	0.12±0.03
I/R + MSCH	7	13.84±1.36	0.43±0.98	6.57±0.97	0.16±0.02	0.12±0.02
**After injury**						
Control	7	12.73±3.34	0.42±0.64	6.24±1.14	0.15±0.04	0.12±0.03
I/R	7	16.99±3.02*	0.42±0.64	7.70±1.19	0.19±0.04	0.15±0.03
I/R + MSCL	7	17.28±4.04*^#^	0.53±0.94	7.70±2.01	0.19±0.04	0.15±0.03
I/R + MSCH	7	19.65±1.60*^&^	0.71±0.91	9.31±1.71*^&^	0.20±0.03	0.16±0.02

Values are mean ± SD. N: number. I/R: ischemia-reperfusion lung injury; MSCL: Treatment with hyoxic MSCs at low-dose (2.5×10^5^); MSCH: Treatment with hypoxia MSCs at high-dose (1×10^6^). *Ppa* and *Ppv* (mmHg): pulmonary arterial and venous pressure, respectively; *Ppc* (mmHg): isogravimetric capillary pressure; *Ra* and *Rv* (cmH2O·min^-1^·ml^-1^): pulmonary arterial and venous resistance.

*P<0.05 compared with control;

^#^P<0.05 *Ppa* of I/R+MSCL compared with I/R or I/R+MSCH;

^&^P<0.05 *Ppa* or *Ppc* of I/R+MSCH compared with I/R or I/R+MSCL.

### Lung weight gain (LWG), the ratio of wet weight/dry weight (W/D), pulmonary capillary filtration coefficient (K_*fc*_), change in airway pressure (ΔP) and white cell count in BALF

Compared to the control group, the I/R group had significantly increased peak airway pressure, K_*fc*_, LWG, W/D and white cell count in BALF. Compared to the I/R group, all of these changes were attenuated in the I/R+MSCL group (Table 2). The I/R+MSCH group had significantly lower K_*fc*_ and white cell count in BALF than the I/R group, but not peak airway pressure, LWG or W/D (Table 2).

**Table 2:** LWG,W/D, WBC, peak airway pressure and K_*fc*_ in various groups.

Group	N	LWG (g)	W/D	WBC count	ΔP airway	Kfc cmH2O · min-1 · ml-1	
						Before	After
Control	7	0.05 ± 0.05	4.88 ± 0.25	140.7 ± 64.1	1.6 ±1.2	0.09± 0.06	0.11 ± 0.10
I/R	7	1.02 ± 0.58*	5.47 ± 0.36*	348.1 ±111.3*	6.5 ± 1.5*	0.10 ± 0.09	0.36 ± 0.21*
I/R + MSCL	7	0.35 ± 0.34*†	4.93 ± 0.25†	198.3 ± 42.0†	4.4 ± 1.4*†	0.08 ± 0.07	0.10 ± 0.09†
I/R + MSCH	7	0.59 ± 0.33*	5.19 ± 0.22*	189.7 ± 87.3†	5.7 ± 2.0*	0.06 ± 0.04	0.10 ± 0.09†

Values are mean ± SD. N: number. LWG: lung weight gain; W/D: lung wet-to-dry weight ratio; WBC: white cell count; ΔP airway: difference between peak airway pressure between baseline and end of experiment; K_*fc*_: pulmonary capillary filtration coefficient.

*P<0.05 compared with control;

^†^P<0.05 compared with I/R.

### Lung pathological change, MPO and lung injury score

**Fig 1:**
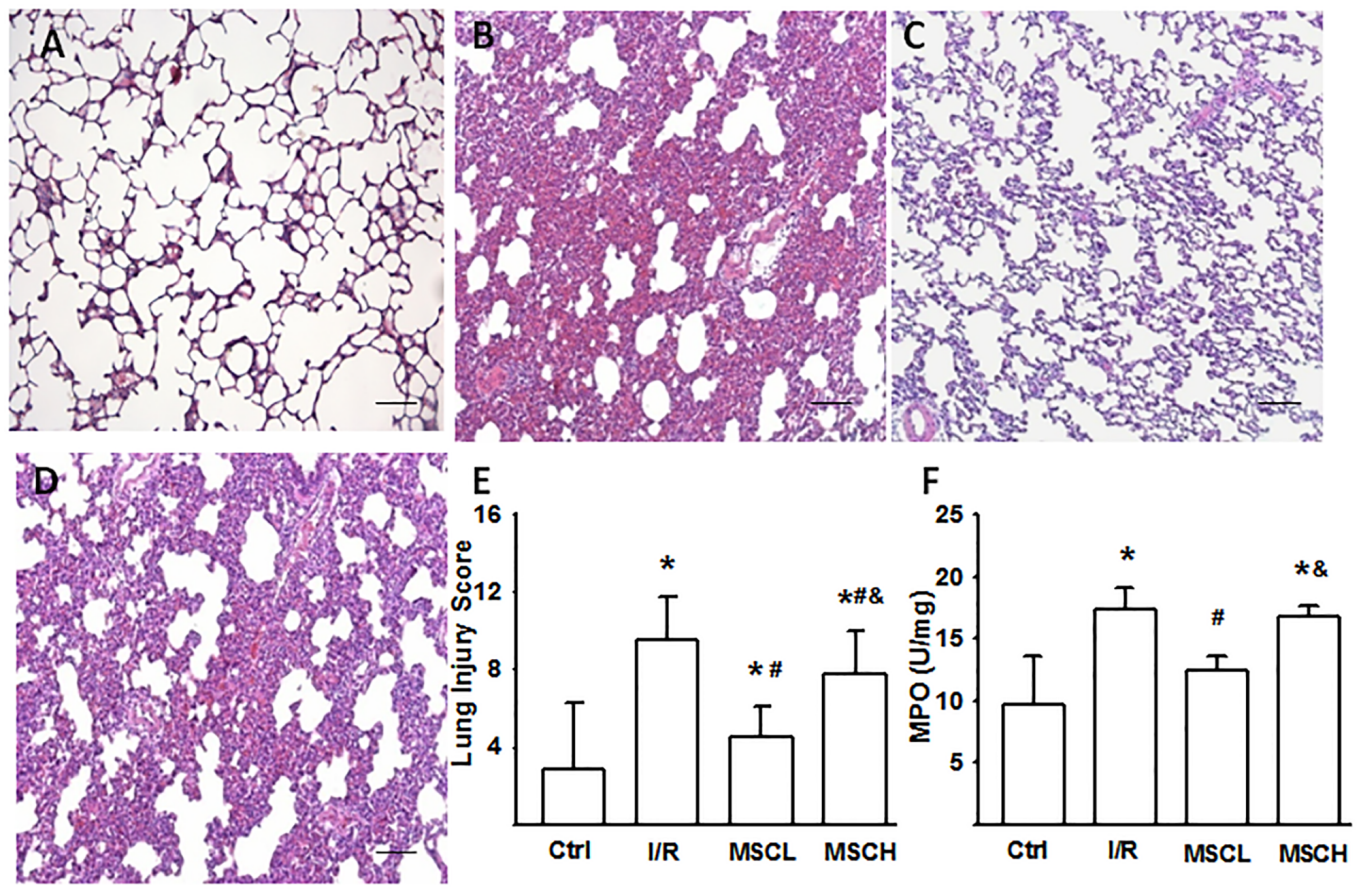
Changes in lung histology and myeloperoxidase (MPO) activity following I/R injury were improved by hypoxic MSC treatment. Lung tissue was stained by HE stain and examined by light microscopy (magnification, ×200; scale bar, 100 μm). Histology of a normal lung (A); lung injury characterized by perivascular edema, alveolar hemorrhage, and leukocyte infiltration in the interstitial and alveolar space after I/R (B); treatment with hypoxic MSCs at low-dose (2.5×10^5^) improved lung histology (C); treatment with high-dose hypoxic MSCs (1×10^6^) had no significant effect on lung histology after lung injury (D). Lung injury scores (E) and MPO activity levels (F) were also shown. N = 7 per group. Error bars represent SEM. MSCL: Treatment with hyoxic MSCs at low-dose (2.5×10^5^); MSCH: Treatment with hypoxia MSCs at high-dose (1×10^6^). *P<0.05 compared with control; ^#^P<0.05 compared with I/R; ^&^P<0.05 compared with I/R+MSCL.

The lung weight and pulmonary capillary filtration coefficient data were further supported by histopathologic analysis showing perivascular edema, intra-alveolar hemorrhage, interstitial and intra-alveolar leukocytic infiltration, and proteinaceous exudates in the I/R group (Fig 1B) compared with the control group (Fig 1A). MPO content, indicating total neutrophil sequestration, was also increased in the I/R group (Fig 1F). Compared to the I/R group, the I/R+MSCL group showed less perivascular edema and inflammatory cell infiltration in lung pathology (Fig 1C), improved lung injury score (Fig 1E) and reduced level of MPO in lung tissue (Fig 1F).

### H_2_O_2_, TBARS, protein carbonyl and glutathione

**Fig 2:**
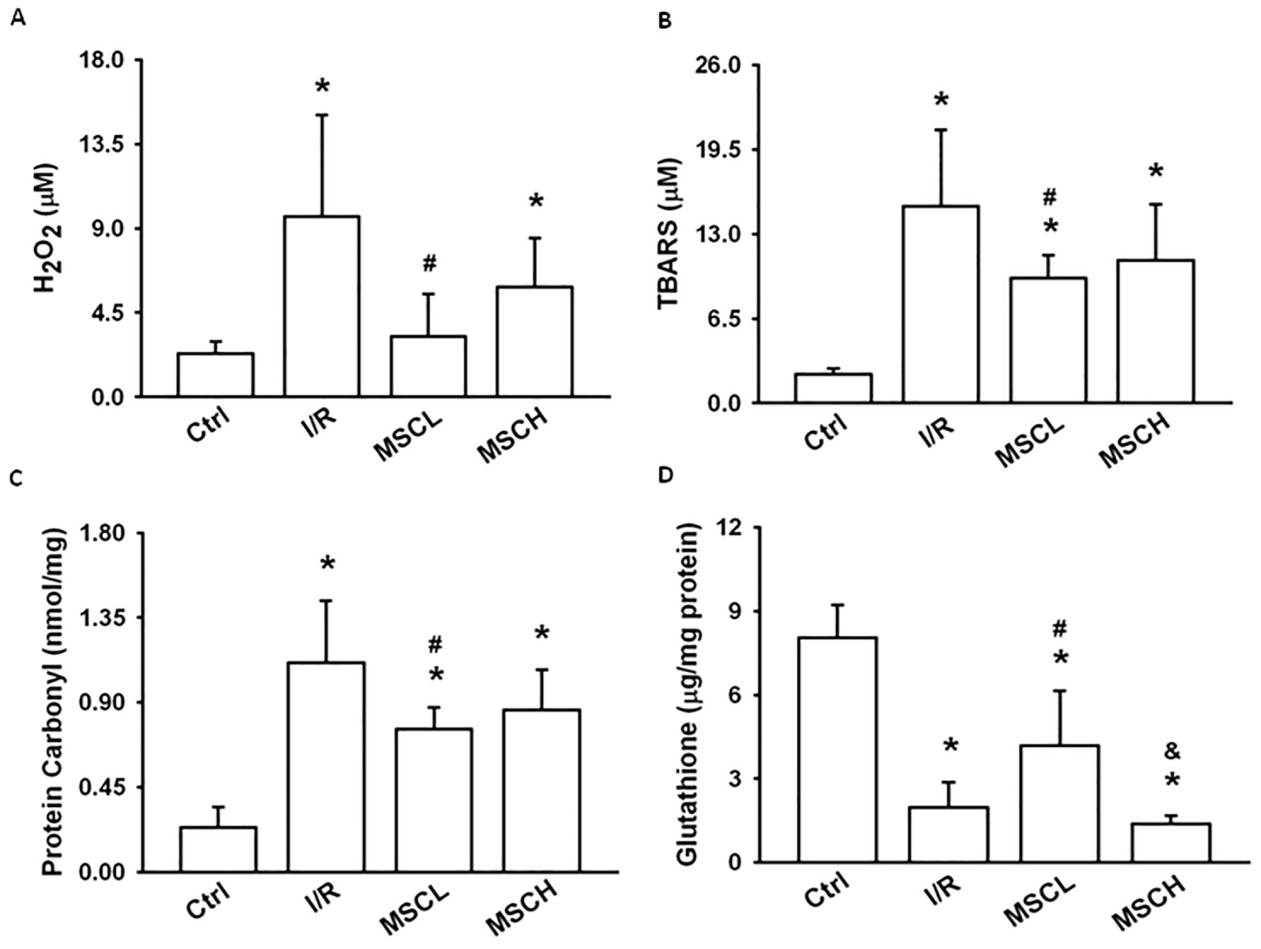
Concentrations of H_2_O_2_, TBARS, and protein carbonyl in BALF and the antioxidant glutathione in lung tissue. The low-dose hypoxic MSC-treated group showed lower levels of H_2_O_2_, TBARS, and protein carbonyl in BALF but higher levels of glutathione in lung tissue. N = 7 per group. Error bars represent SEM. MSCL: Treatment with hyoxic MSCs at low-dose (2.5×10^5^); MSCH: Treatment with hypoxia MSCs at high-dose (1×10^6^). *P<0.05 compared with control groups; ^#^P<0.05 compared with I/R groups; ^&^P<0.05 compared with I/R+MSCL. BALF = bronchoalveolar lavage fluid.

Compared to the control group, H_2_O_2_, TBARS and protein carbonyl were higher in BALF, however glutathione was lower in the lung tissue of the I/R group (Fig 2A–2D). In addition, compared to the I/R group, H_2_O_2_, TBARS and protein carbonyl were lower in BALF, however glutathione was higher in the lung tissue of the I/R+MSCL group (Fig 2A–2D).

### TNF-α, IL-1β, MIP-2, IL-10 and prostaglandin E2

**Fig 3:**
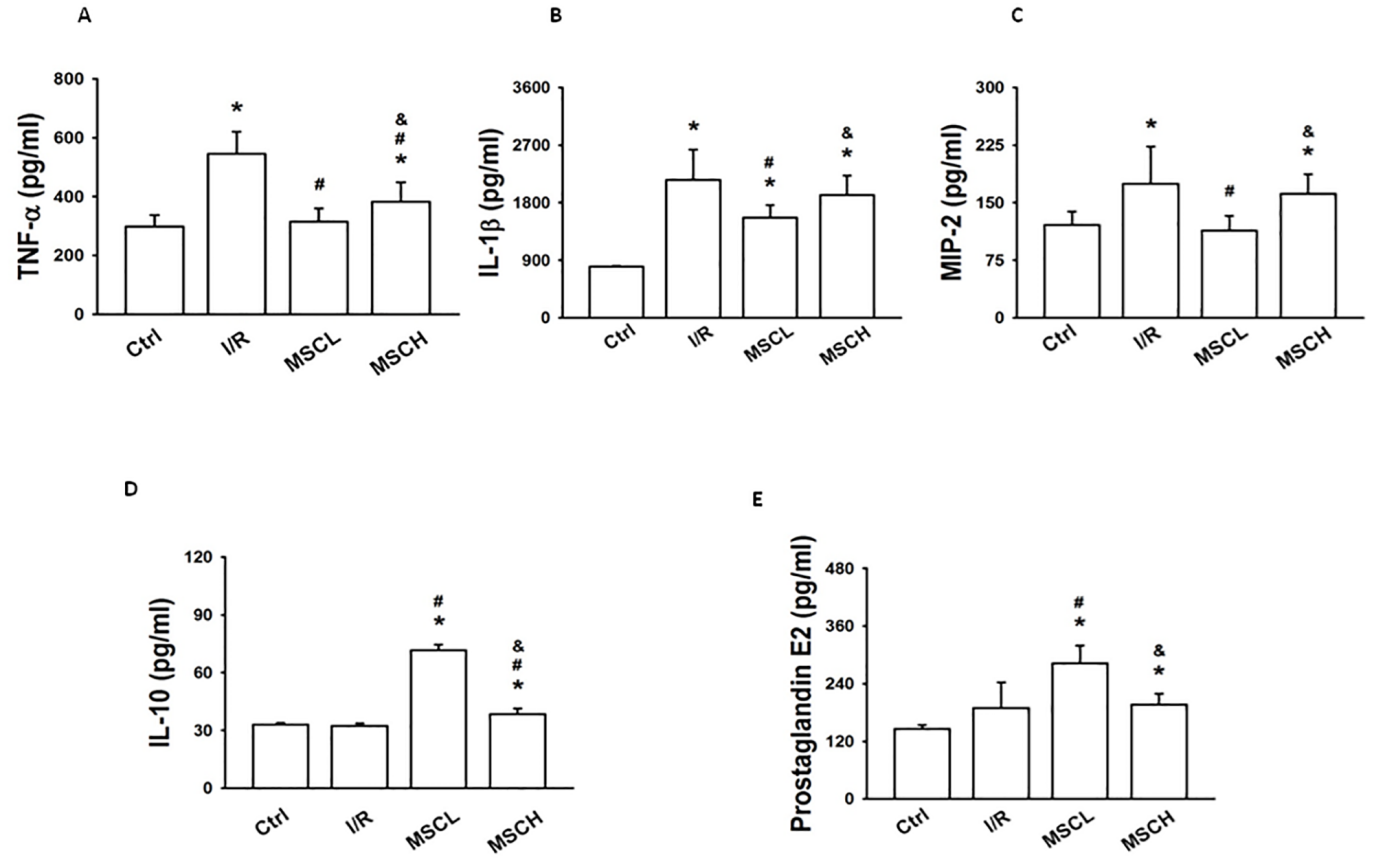
The levels of TNF-α, IL-1β, MIP-2, IL-10, and prostaglandin E2 in BALF. Low-dose hypoxic MSC treatment down-regulated TNF-α, IL-1β, and MIP-2 expression levels but up-regulated the levels of IL-10 and prostaglandin E2 in BALF. N = 7 per group. Error bars represent SEM. MSCL: Treatment with hyoxic MSCs at low-dose (2.5×10^5^); MSCH: Treatment with hypoxia MSCs at high-dose (1×10^6^). *P<0.05 compared with control groups; ^#^P<0.05 compared with I/R groups; ^&^P<0.05 compared with I/R+MSCL. BALF = bronchoalveolar lavage fluid.

The levels of TNF-α, IL-1β and MIP-2 in BALF of the I/R group were higher than those in the control group (Fig 3A–3C). Compared to the I/R group, levels of TNF-α, IL-1β and MIP-2 were lower but levels of IL-10 and prostaglandin E2 were higher in BALF of the I/R+MSCL group (Fig 3A–3E). In addition, the level of TNF-α was lower but the level of IL-10 was higher in the I/R+MSCH group than in the I/R group (Fig 3A and 3D).

### Intercellular adhesion molecule 1 (ICAM-1) and vascular cell adhesion molecule 1 (VCAM-1)

**Fig 4:**
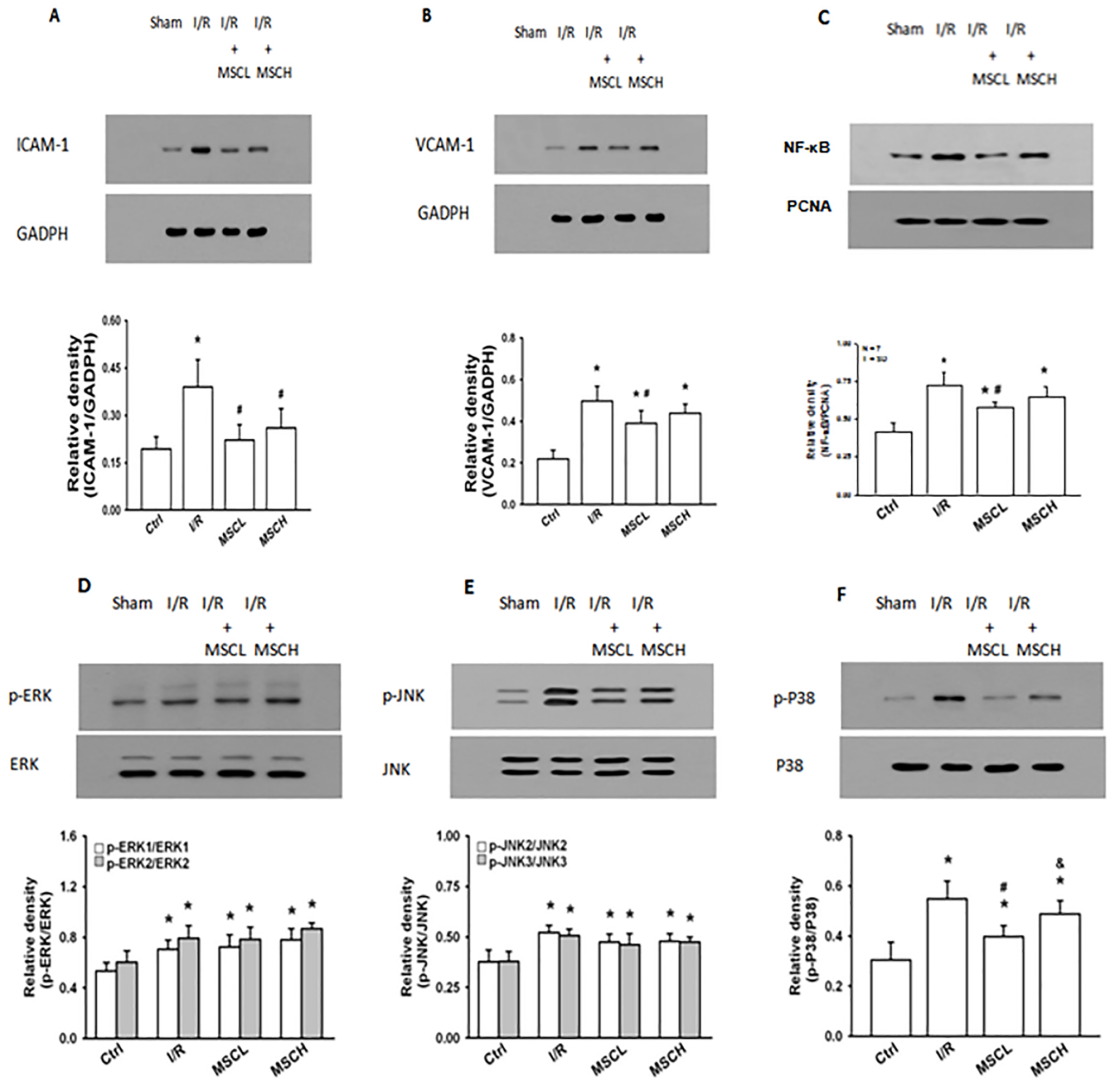
Expression of adhesion and signaling transduction molecules in the lung tissue. Expression levels of ICAM-1 and VACM-1 in lung tissue (A and B). Activation of nuclear factor kappa B (NF-κB) and the mitogen-activated protein kinases (MAPKs) extracellular signal-regulated protein kinase (ERK), N-terminal kinases (JNK), and P38 in the lung tissue as detected by specific antibodies (C-F). Low-dose hypoxic MSC treatment reduced the levels of adhesion molecules and decreased the activation of NF-κB and P38 MAPK following I/R lung injury. N = 7 per group. Error bars represent SEM. MSCL: Treatment with hypoxia MSCs at low-dose (2.5×10^5^); MSCH: Treatment with hypoxia MSCs at high-dose (1×10^6^). *P<0.05 compared with control groups; ^#^P<0.05 compared with I/R groups; ^&^P<0.05 compared with I/R+MSCL.

The ICAM-1 and VCAM-1 contents in lung tissue were higher in the I/R group than in the control group, but lower in the I/R+MSCL group. However, the level of ICAM-1 but not VCAM-1 was significantly lower in the I/R+MSCH group compared to the I/R group (Fig 4A and 4B).

### Nuclear factor kappa B (NF-κB) and mitogen-activated protein kinase (MAPK)

The expression of NF-κB, extracellular signal-regulated protein kinase (ERK), c-Jun N-terminal kinases (JNK) and P38 MAPK were upregulated in lung tissue of the I/R group (Fig 4C–4F). Compared to the I/R group, treatment with low-dose MSCs (2.5×10^5^) induced down-regulation of the NF-κB and P38 MAPK pathways, but not ERK and JNK pathways (Fig 4C–4F).

### Caspase-3, Bcl-2, mitochondrial and cytosolic cytochrome C, and apoptotic index in lung tissue

**Fig 5:**
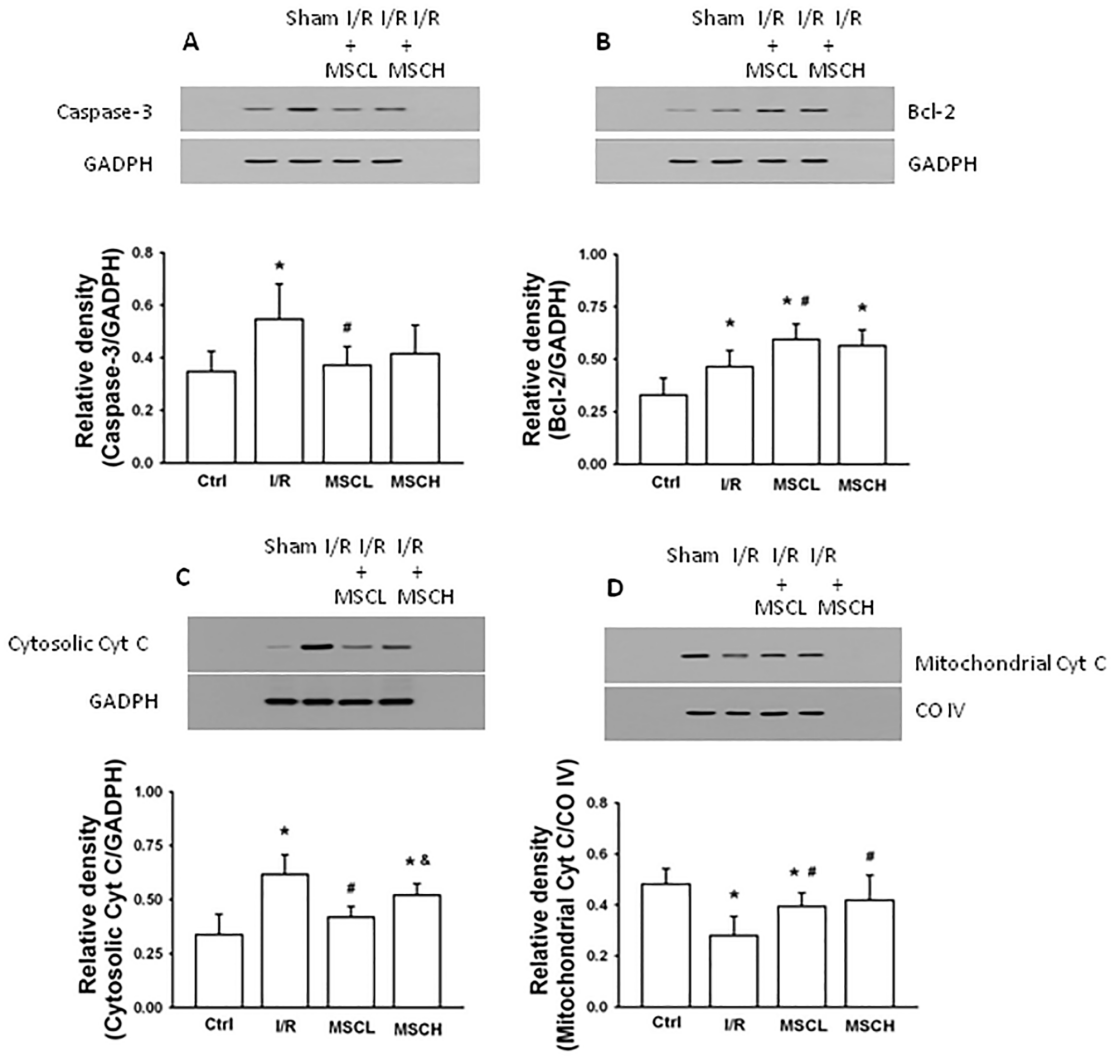
Measurement of pro-apoptotic and anti-apoptotic biomarkers in lung tissue. Expression of the apoptotic markers caspase-3 (A), Bcl-2 (B), and cytosolic and mitochondrial cytochrome C (C-D) in lung tissue were measured by western blotting with specific antibodies. Treatment with low-dose hypoxic MSCs inhibited caspase-3 and cytosolic cytochrome C and restored Bcl-2 and mitochondrial cytochrome C levels following I/R lung injury. N = 7 per group. Error bars represent SEM. MSCL: Treatment with hyoxic MSCs at low-dose (2.5×10^5^); MSCH: Treatment with hypoxia MSCs at high-dose (1×10^6^). *P<0.05 compared with control groups; ^#^P<0.05 compared with I/R groups; ^&^P<0.05 compared with I/R+MSCL.

**Fig 6:**
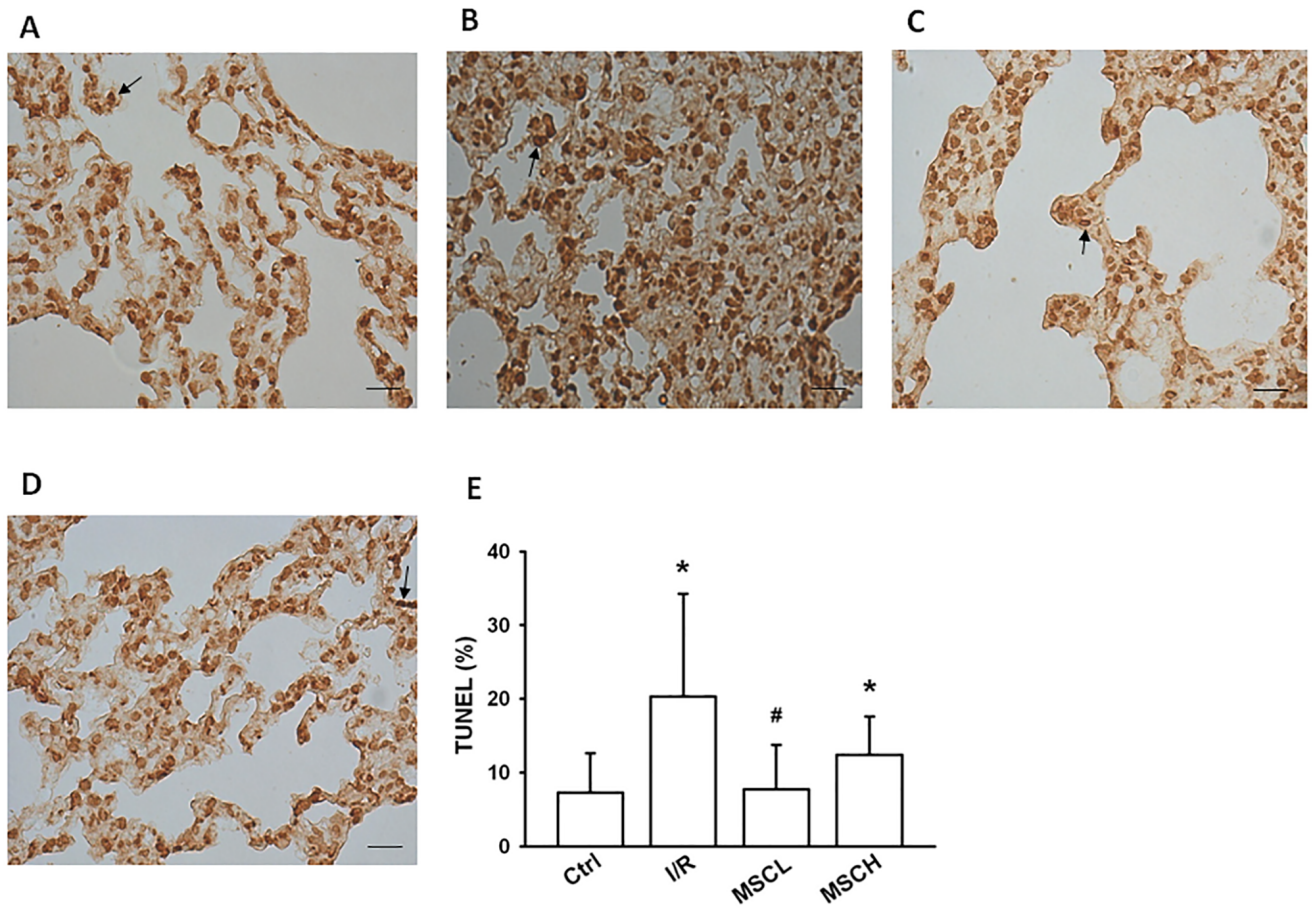
Quantitation of lung apoptosis in lung tissue by TUNEL staining. Representative micrographs of TUNEL-stained paraffin-embedded lung sections (magnification, ×400; scale bar, 20 μm) of the control (A), I/R (B), low-dose hypoxic MSC-treated (C), and high-dose hypoxic MSC-treated (D) groups. Quantitation of the TUNEL-positive cells in lung tissues (E). Low-dose hypoxic MSCs attenuatd apoptosis, as evidenced by TUNEL-stained lung tissue and apoptotic scoring of lung tissue. Apoptotic cellular nuclei were stained dark brown. The black arrow in each panel is an apoptotic nucleus (A-D). N = 7 per group. Error bars represent SEM. MSCL: Treatment with hyoxic MSCs at low-dose (2.5×10^5^); MSCH: Treatment with hypoxia MSCs at high-dose (1×10^6^). *P<0.05 compared with control group; ^#^P<0.05 compared with I/R group.

Compared to the control group, up-regulation of caspase-3 and Bcl-2 in lung tissue was found in the I/R group (Fig 5A and 5B). Futhermore, the level of cytosolic cytochrome C was higher but the level of mitochondrial cytochrome C was lower in lung tissue of the I/R group (Fig 5C and 5D). Compared to the I/R group, down-regulation of caspase-3 but up-regulation of Bcl-2 was noted in the I/R+MSCL group (Fig 5A and 5B). In addition, the level of mitochondrial cytochrome C recovered but the level of cytosolic cytochrome C decreased in the I/R+MSCL group (Fig 5C and 5D). More apoptotic cells (as evidenced by brown nuclei in TUNEL staining) (Fig 6B) were noted in the I/R group compared to the control group (Fig 6A). Furthermore, the I/R+MSCL group, but not the I/R+MSCH group, demonstrated significantly reduced lung apoptosis with a lower apoptotic score compared to the I/R group (Fig 6C–6E).

### Pulmonary emboli

**Fig 7:**
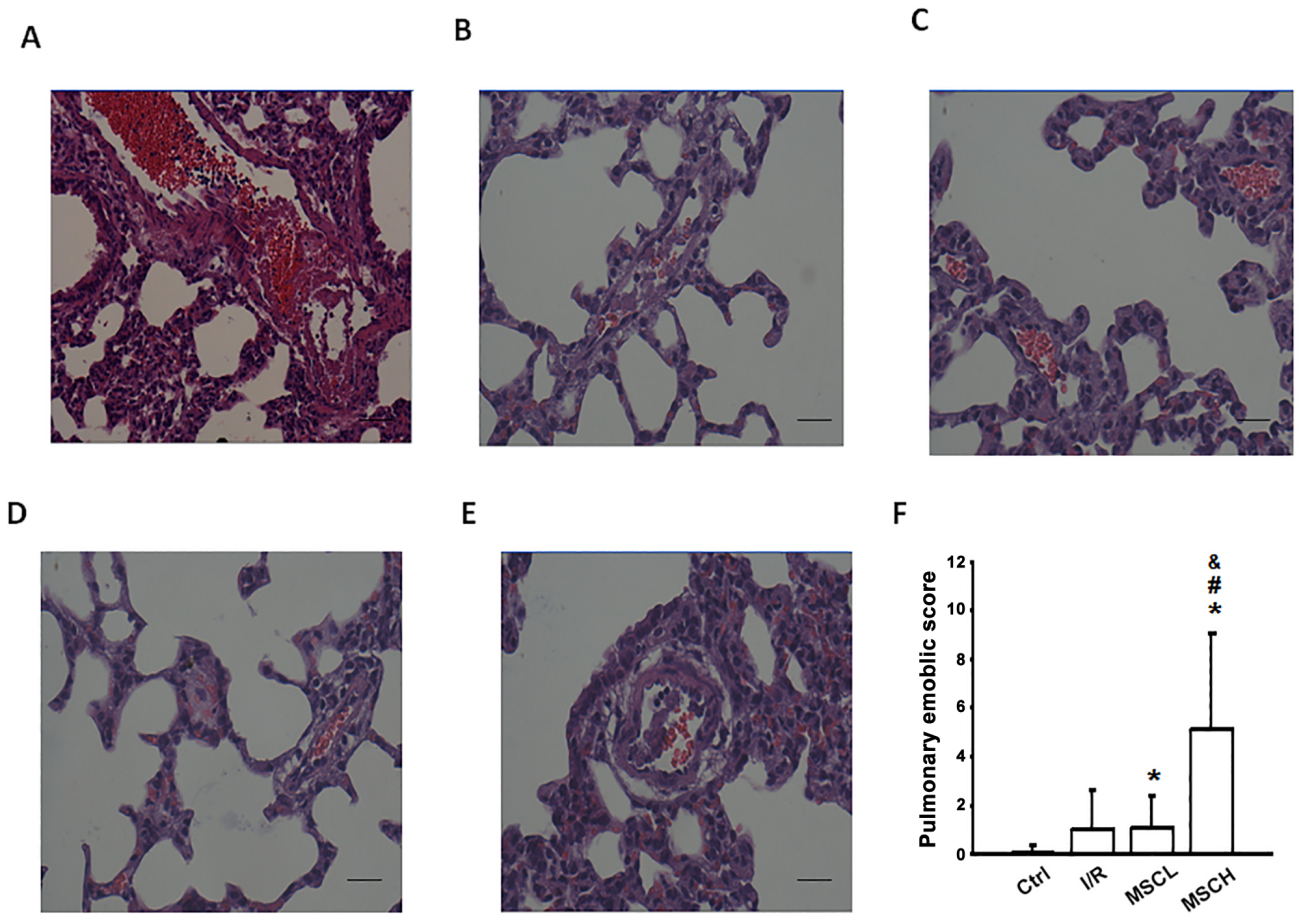
Infusion of high-dose hypoxic MSCs induced more thrombosis and pulmonary emboli. Pulmonary emboli were observed in the pulmonary arteries of various sizes in the I/R+MSCH group, including a large pulmonary artery (A), medium pulmonary artery (E), and small pulmonary arterioles (B, C, D) (magnification, ×400; scale bar, 20 μm). The pulmonary embolic scores for the four groups are displayed in (F). N = 7 per group. Error bars represent SEM. MSCL: Treatment with hyoxic MSCs at low-dose (2.5×10^5^); MSCH: Treatment with hypoxia MSCs at high-dose (1×10^6^). *P<0.05 compared with control groups; ^#^P<0.05 compared with I/R groups; ^&^P<0.05 compared with I/R+MSCL.

Pulmonary embolism and thrombosis were found in various sizes of pulmonary artery in the I/R+MSCH group, including the large pulmonary artery (Fig 7A), medium pulmonary artery (Fig 7E) and small pulmonary arteriole (Fig 7B–7D), and a higher embolic score was found in both the I/R+MSCL and I/R+MSCH groups compared with the control group. Moreover, the pulmonary embolic score after treatment with a high dose of MSCs (1×10^6^) was markedly higher than in the other groups (Fig 7F). These results suggest that a higher dose of hypoxic MSCs was prone to forming aggregates in the pulmonary microcirculation and exacerbate post-transplantation embolic events.

### Hypoxic MSCs infiltrate into extravascular regions of lung

**Fig 8:**
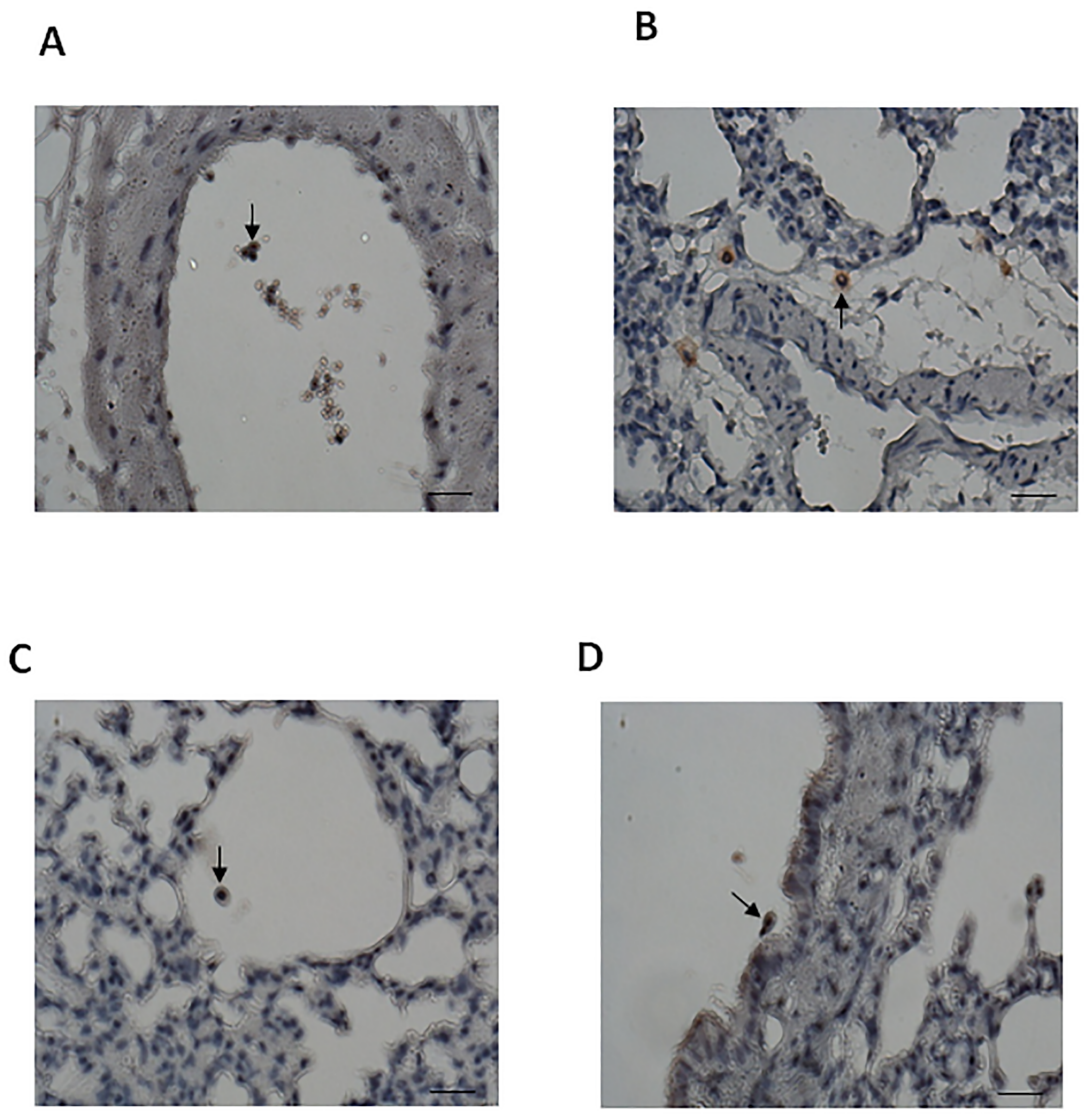
MSCs quickly migrated into the extravascular regions of the lung. The nuclei of the MSCs were stained dark brown, and the figure shows MSCs in the extravascular regions of the lung. MSCs were found in pulmonary vessels (A), and MSCs adhered to other cells might be involved in crosstalk. MSCs also migrated into the pulmonary interstitium (B), alveoli (C), and bronchial tree (D) (magnification, ×400; scale bar, 20 μm).

MSCs were tracked by BrdU incorporation followed by immunostaining with an antibody against BrdU and the use of DAB as substrate. Brown-colored hypoxic MSCs were found in pulmonary vessels (Fig 8A), interstitial (Fig 8B) and alveolar spaces (Fig 8C) and bronchial trees (Fig 8D) which reflected that MSCs infiltrated into all compartments of the lung via the pulmonary circulation in 2 hours. Intriguingly, some MSCs were shown to be adhered to other inflammatory or parenchymal structural cells.

## Discussion

I/R injury strongly impacts the clinical outcomes of various critical medical interventions, including organ transplantation, resuscitation, and major cardiac operations. However, there is no effective therapy. MSC-based cell therapies have been widely evaluated in clinical trials as potential therapeutic approaches for various diseases, and we previously showed that hypoxia preconditioning can promote MSCs to immune-privileged status, enhance their paracrine effects, and increased their mobilization and homing abilities [24, 26–27]. Likewise, a growing body of evidence suggests that hypoxia-preconditioned MSCs have superior beneficial effects compared to normoxic MSCs in animal models of bleomycin-induced pulmonary fibrosis, myocardial ischemia, acute kidney injury, and limb ischemia [26, 28–30]. Our study is the first to administrate hypoxic MSCs to treat I/R lung injury in a pre-clinical animal model. The major findings of this study are: (1) hypoxic MSCs delivered intravascularly quickly migrate into the interstitium, alveolar spaces, and bronchial tree; (2) administration of low-dose hypoxic MSC attenuated I/R, while high-dose infusion induced considerable pulmonary embolism, leading to increased pulmonary arterial pressure; (3) hypoxic MSCs attenuated I/R lung injury by decreasing capillary leakage, inflammation, oxidative stress, and apoptosis.

### Hypoxic MSCs ameliorated lung function and architectural damage

I/R induced pulmonary edema (increased lung weight and wet/dry lung weight ratio) and pulmonary capillary leakage (increased K_*fc*_) which was attenuated by treatment with hypoxic MSCs. Lung compliance in the low-dose MSC-treated group was higher than that in the I/R group, as reflected by the decrease in peak airway pressure. Furthermore, the group treated with low-dose hypoxic MSCs showed a lower lung injury score (less inflammatory cell infiltrates and edema). The pulmonary physiological and pathological changes associated with I/R were consistent with those observed in previous studies [2, 5–6]. Here we demonstrated, for the first time, that an optimal dose of hypoxic MSCs attenuated I/R-induced pathological changes and changes in pulmonary function.

### Hypoxic MSCs reduced oxidative stress

We showed that the levels of protein carbonyl, TBARS, and H_2_O_2_, which reflect ROS content, in BALF were higher following I/R lung injury and these results are consistent with those of a previous study [2]. These oxidative products were decreased in the low-dose hypoxic MSC-treated group, indicating that hypoxic MSCs can suppress the oxidative stress evoked by I/R lung injury. Moreover, our data showed that anti-oxidant capacity (glutathione levels) was increased by low-dose hypoxic MSCs treatment when compared to the levels in the I/R group. This finding showed hypoxic MSCs can exert anti-oxidant effects by restoring the oxidant scavenging enzymes.

### Anti-inflammatory effects of hypoxic MSCs

The results showed that I/R initially induced the alveolar macrophages to secrete TNF-α and IL-1β to prime the inflammatory response. The, numerous neutrophils were attracted by MIP-2 and infiltrated and attacked the graft [1]. Our results also showed that I/R induced upregulation of pro-inflammatory cytokines. Furthermore, inflammatory cells were recruited to and activated in I/R lung injury, as reflected by higher MIP-2 and MPO expression, increased inflammatory cell counts in BALF, and greater inflammatory cell infiltration in the lung tissue. These results are consistent with our previous study [2]. Bone marrow stromal cells have been shown to modulate the activation of host macrophages through prostaglandin E2 to promote the secretion of more IL-10 and protect against sepsis in mice [31]. Likewise, IL-10-engineered MSCs prevented I/R lung injury in an animal model [32]. Our data showed that treatment with low-dose hypoxic MSCs decreased the expression levels of various pro-inflammatory cytokines, such as TNF-α, IL-1β, and MIP-2, and promoted the secretion of the anti-inflammatory molecules IL-10 and prostaglandin E2. Treatment with low-dose hypoxic MSCs also reduced lung neutrophil recruitment, which was reflected by decreased inflammatory cell infiltration and MPO levels in the lung tissue and lower white cell counts in BALF. The signaling pathways downstream of the inflammatory response to I/R are mediated by phosphorylation of MAP kinases (p-ERK, p-JNK, p-P38 MAPK) and translocation of NF-kB into the nucleus [2]. In our model, only P38 MAPK and NF-κB activation were inhibited by administration of low-dose hypoxic MSCs. This finding indicates that hypoxic MSCs attenuated I/R lung injury through the P38 MAPK and NF-κB pathways.

The expression of ICAM-1, and VCAM-1 were increased following I/R but were attenuated by low-dose hypoxic MSCs treatment. We speculate that the lung-protective effects of hypoxic MSCs might be related to suppression of the cellular crosstalk between leukocytes and the structural cells in the lung as a result of decreased expression of epithelial and endothelial surface adhesion molecules or the co-stimulatory molecules ICAM-1 and VCAM-1. Collectively, these results showed that hypoxic MSCs have anti-inflammatory effect on I/R lung injury. However, the exact mechanisms by which hypoxic MSCs protect against I/R-induced lung injury have yet to be elucidated. Previous investigations using a model of acute lung injury induced by endotoxin or I/R injury suggested that these paracrine effects were mediated by soluble factors released from MSCs (different from the MSCs used in this study) [19, 31–35]; however, the target of the MSCs is unknown.

### Anti-apoptotic effects of hypoxic MSCs

P38 MAPK is a protein kinase that is involved in cell differentiation and apoptois in response to inflammatory cytokines, and the transcription factor NF-κB regulaties apoptosis in diverse cell types. In our study, we demonstrated that low-dose hypoxic MSCs decreased P38 MAPK phosphorylation and activation of NF-κB. The apoptotic pathways is initiated by TNF-α and facilitated by caspase-3, whereas Bcl-2 inhibits the release of cytochrome C from the mitochondria to cytosol, which is a pivotal anti-apoptotic signal in the apoptosis cascade. The hypoxic MSCs inhibited activation of TNF-α and caspase-3 and increased the levels of Bcl-2 and mitochondrial cytochrome C following I/R lung injury. Furthermore, hypoxic MSCs improved the apoptosis score, as reflected by TUNEL staining. Thus, administration of low-dose hypoxic MSCs decreased lung apoptosis, which appeared to be mediated by modulation of pro-apoptotic and anti-apoptotic pathways. This decrease in apoptosis might also be due to reduced oxidative stress and cytokine responses since the inflammatory mediators are also involved in activation of the caspase cascades related to apoptosis. Our findings support the results of previous studies that showed markedly reduced acute I/R lung injury after administration of different MSCs (adipose-derived MSCs) occurred through anti-inflammation and anti-apoposis activities [35–36].

### Pulmonary embolism induced by infusion of hypoxic MSCs

We showed that pulmonary embolism occurred after infusion of hypoxic MSCs, especially at a high dose. We postulate that when a high concentration of hypoxic MSCs is infused under stress conditions, the hypoxic MSCs adhere to each other, resulting in vascular obstruction. Thus, high dose MSC treatment caused significant increase in pulmonary arterial and capillary pressure to impede the delivery of stem cells. Similarly, Cui et al. [37] reported cerebral embolic events after intra-arterial delivery of bone marrow MSCs in rats subjected to a middle cerebral artery occlusion procedure. They concluded that these post-transplantation complications were related to cell dose and infusion velocity [37]. Intravascular administration of MSCs is the most commonly used delivery route for MSC-based cell therapies in clinical trials. Nevertheless, accumulating clinical evidence has shown that intravascular delivery of MSCs can cause substantial vascular obstruction events [38]. Based on these results, optimization of the injected dose of MSCs is a critical concern for the clinical safety of MSC-based cell therapies.

### Hypoxic MSCs quickly migrate from the pulmonary artery into the lung tissue

We demonstrated that hypoxic MSCs delivered intravascularly migrate into the extravascular regions of the lung, including the interstitial and intra-alveolar spaces and bronchial trees. A previous study reported that MSCs administered intravenously migrated into the lung; however, which part of the lung tissue was not reported [39]. Notably, we found that hypoxic MSCs also adhere to inflammatory cells and structural cells, and these cell-to-cell interactions suggest possible crosstalk, resulting in the release of mediators that function in a paracrine manner to protect against I/R lung injury.

There are some limitations to this study. First, we used an isolated perfused rat lung model to minimize any hemodynamic effects on lung injury; thus, the model excludes cross communication with other organ systems that may occur *in vivo*. Second, our original plan was to investigate the dose-dependent response to stem cell therapy in I/R lung injury. Thus, we doubled and halved the effective therapeutic dose that Rojas et al. [15] used in an animal model of lung injury and repair. Incidentally, we observed that the administration of high-dose hypoxic MSCs induced more severe pulmonary embolism complications, leading to increased vascular obstruction. Therefore, the mechanisms underlying pulmonary embolism need to be studied to determine the optimal dose of MSCs for use clinical practice. Third, the beneficial actions of hypoxic MSCs are mostly mediated via a paracrine effect. However, Chailakyan et al. [40] reported that administration of hypoxic MSCs had better effects than conditioned medium in a model of ALI. They attributed the advantages of cell transplantation to cell-to-cell interactions between stem cells, the endothelium, and the pulmonary interstitium. The effects of conditioned medium on I/R lung injury remain to be investigated.

## Conclusions

Hypoxia MSCs attenuate I/R lung injury through anti-oxidant, anti-inflammatory, and anti-apoptotic mechanisms, and these beneficial effects are partly mediated via the P38 MAPK and NF-κB pathways. The effects of hypoxic MSCs on I/R lung injury were dependent on the number of hypoxia MSCs infused, as a high dose of hypoxic MSCs caused pulmonary emboli and pulmonary hypertension, which blocked the beneficial effects of MSCs treatment. Notably, hypoxia MSCs can quickly migrate into the extravascular spaces in the injured lung and adhere to inflammatory and structure cells. We hypothesize that there may be crosstalk between the infused hypoxic MSCs and other cells, which then release mediators to attenuate I/R in a paracrine manner.

## Supporting information

S1 Fig Hypoxic culture maintained expansion efficiency at low density. SD rat MSCs at passage 0 were photographed at (A) 3 days and (B) 9 days of culture. The cells were recovered and subcultured at the density of 100 cells/cm^2^ and subcultured at 9 days with the same density. (C) The fold increase in cell number and (D) population doubling for each passage were calculated. Pn = passage number. Bar = 40mm.
(TIF)

S2 Fig Cell surface marker profile of MSCs expanded under hypoxic conditions. MSCs at passage 6 were used for flow cytometric analysis. Matched isotype IgG controls are shown as non-shaded areas.
(TIF)

S3 Fig MSCs which expended under hypoxic conditions maintained differentiation potential. MSCs at passage 6 were induced for osteogenesis, adipogenesis and chondrogenesis for 21 days. MSCs induced without (CTR) or with osteogenesis, adipogenesis and chondrogenesis were assayed for Alzarin Red S (ARS), Oil-Red O, and Alcian blue and immunostaining for Type II collagen, respectively. Bar = 40 mm.
(TIF)

S1 File Detailed method description of the animal experiments. (DOC)

## Abbreviations

Bcl-2: B-cell lymphoma 2
ERK: extracellular signal-regulated kinases
ICAM-1: intercellular adhesion molecule 1
IL-1β: interleukin-1β
I/R: ischemia/reperfusion injury
JNK: Jun N-terminal kinases
K_*fc*_: pulmonary capillary filtration coefficient
LWG: lung weight gain
MIP-2: macrophage inflammatory protein 2
MPO: myeloperoxidase
MSCs: mesenchymal stem cells
NF-kB: nuclear factor kappa-κB
P_*pa*_: pulmonary arterial pressure
P38 MAPK: P38 mitogen-activated protein kinase
TNF-α: tumor necrosis factor-α
VCAM-1: vascular cell adhesion protein 1
