# Supplementary figures and images for: Hypoxia-preconditioned mesenchymal stem cells ameliorate ischemia/reperfusion-induced lung injury
Source: PLoS One. 2017 Nov 8;12(11):e0187637. doi: 10.1371/journal.pone.0187637 (PMC5678873; doi:10.1371/journal.pone.0187637)

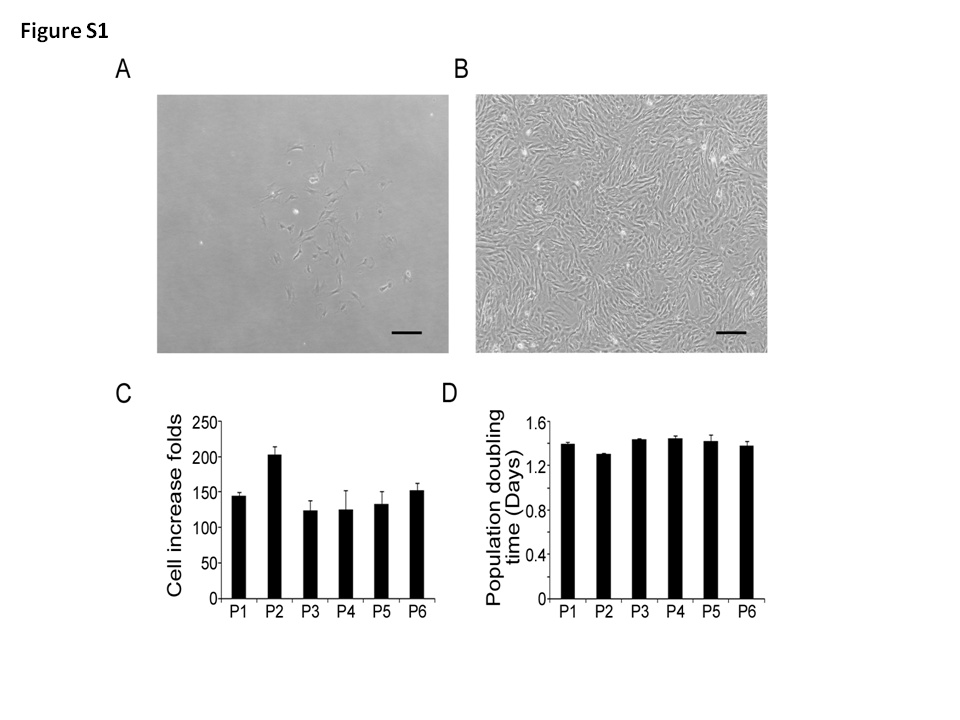

Supplement: S1 Fig — SD rat MSCs at passage 0 were photographed at (A) 3 days and (B) 9 days of culture. The cells were recovered and subcultured at the density of 100 cells/cm2 and subcultured at 9 days with the same density. (C) The fold increase in cell number and (D) population doubling for each passage were calculated. Pn = passage number. Bar = 40mm. (TIF) [file pone.0187637.s001.tif]

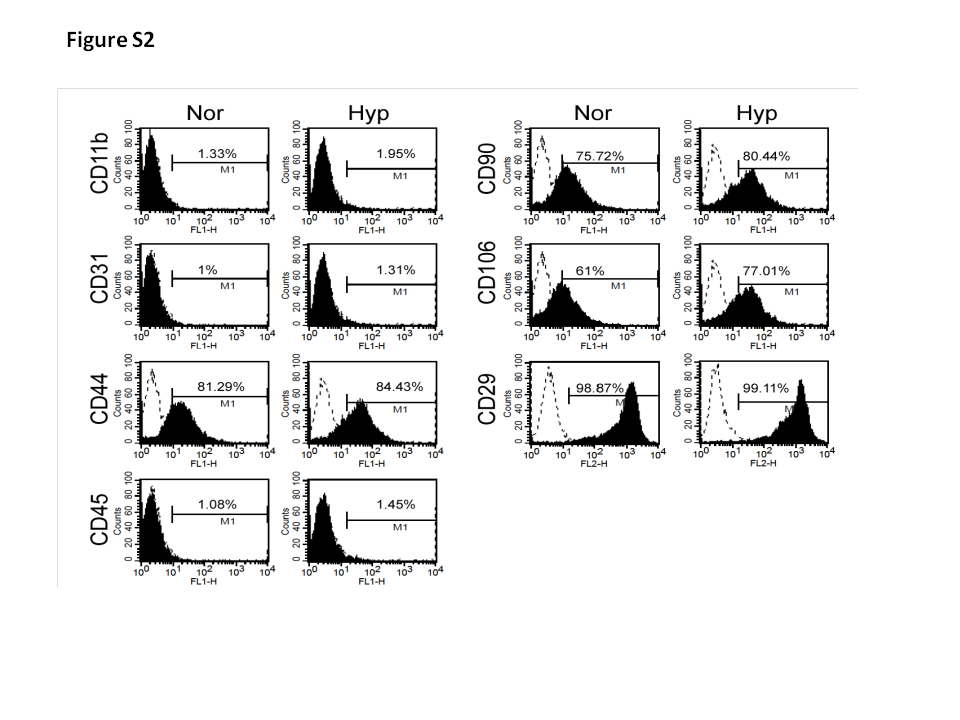

Supplement: S2 Fig — MSCs at passage 6 were used for flow cytometric analysis. Matched isotype IgG controls are shown as non-shaded areas. (TIF) [file pone.0187637.s002.tif]

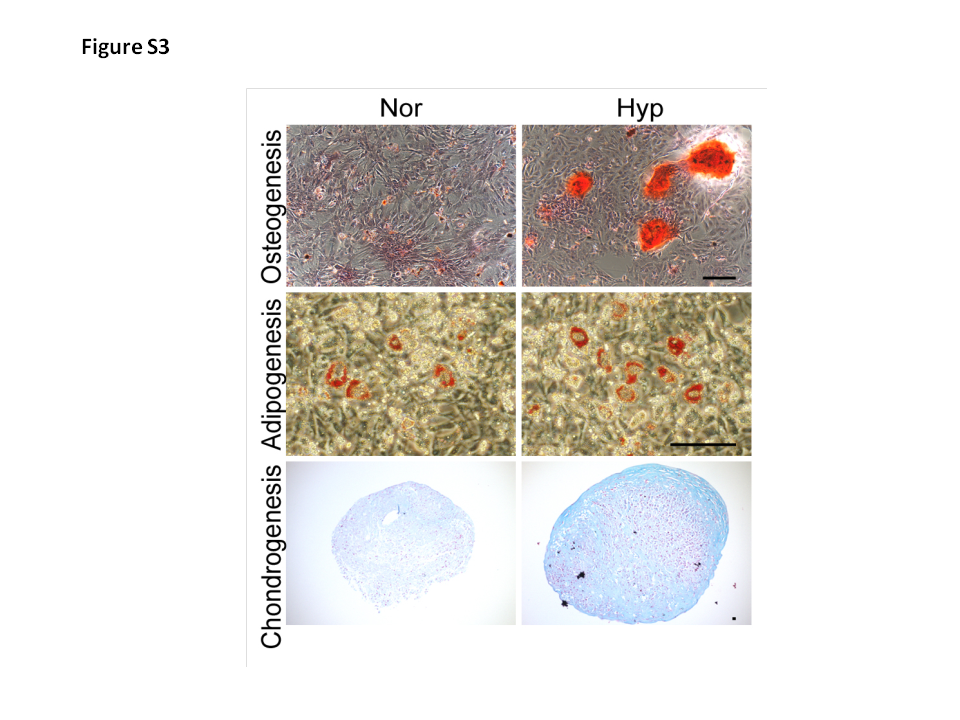

Supplement: S3 Fig — MSCs at passage 6 were induced for osteogenesis, adipogenesis and chondrogenesis for 21 days. MSCs induced without (CTR) or with osteogenesis, adipogenesis and chondrogenesis were assayed for Alzarin Red S (ARS), Oil-Red O, and Alcian blue and immunostaining for Type II collagen, respectively. Bar = 40 mm. (TIF) [file pone.0187637.s003.tif]
