# Supplementary material for: Hypoxia-preconditioned mesenchymal stem cells ameliorate ischemia/reperfusion-induced lung injury
Source: PLoS One. 2017 Nov 8;12(11):e0187637. doi: 10.1371/journal.pone.0187637 (PMC5678873; doi:10.1371/journal.pone.0187637)
Supplement: S1 File — (DOC) [file pone.0187637.s004.doc]

**Supporting Information**

**S1 File. Detailed method description of the animal experimentsMETHODS**

**Bone marrow cell culture**

The study protocol was approved by our institutional board for animal care and use of Taipei Veterans General Hospital, Taiwan. We have performed the experiments in according to the National Institutes of Health Guidelines on the Use of Laboratory Animals. Male Sprague-Dawley rats weighing 250-350 grams were anesthetized with intraperitoneal injection of sodium pentobarbital. Mononuclear cells were isolated from the bone marrow by density gradient centrifugation method, and suspended in complete medium [CCM: α-MEM (α-minimal essential medium; Gibco-BRL, Gaithersburg, MD), supplemented with 16.6% fetal bovine serum (FBS), 100 units/mL penicillin, 100 μg/mL streptomycin, and 2 mM L-glutamine]. After 24 h of the initial culture, non-adherent cells were removed. The culture reaches 65–70% confluence within 6-8 days and reaches subconfluence at 10 days (Passage 0). The cells were seeded at 50 cells per cm^2^ in CCM. Flow cytometry analysis indicated that the majority of cells expressed the MSC surface markers CD29, CD44H, CD54, CD73 and CD90, but only few cells expressed CD31, CD34 and CD45 .The surface antigens and differentiation potential of the cells were well characterized to ensure that they were BM-MSCs. Cells from the 4th passage were used in the subsequent assays. For hypoxic culture, cells were cultured in a gas mixture composed of 94% N_2_, 5%CO_2_, and 1% O_2_. The hypoxic conditions were monitored and maintained by an automatic system.

**Measurement of microvascular permeability**

Pulmonary capillary filtration coefficient (K *_fc_*) was used as an index of microvascular permeability to water. The K_fc_ was measured by using the method described previously [1-3]. Briefly, after an isogravimetric period, P_pv_ was rapidly elevated to 6–8 cmH_2_O for 15 min. The increase in lung weight was recorded, and a characteristic rapid weight gain (vascular filling) was followed by a slower rate of weight gain. The rate of weight change (DWt/Dt) during the 6- to 14-min interval was analyzed using linear regression of the log10-transformed rates of weight changes per minute. The initial rate of weight gain was calculated by using extrapolation of DWt/Dt to time 0. K *_fc_* was calculated by dividing DWt/Dt at time 0 by the changes in P_pc_ that occurred after venous outflow pressure was increased, normalized using the baseline wet lung weight, and expressed as mL/min/cm H_2_O per 100 g of lung tissue.

**Isolation of rat lung mitochondria**

Lung tissue was minced on ice, resuspended in 50 ml of MSH buffer (210 mM mannitol/70 mM sucrose/5 mM Hepes, pH 7.5) supplemented with 1 mM EDTA, and homogenized with a glass Dounce homogenizer and Teflon pestle. Homogenates were centrifuged at 600 *g* for 8 min at 4°C. The supernatant was decanted and recentrifuged at 5,500 *g* for 15 min to form a mitochondrial pellet that was resuspended in MSH buffer without EDTA and centrifuged again at 5,500 *g* for 15 min. The final mitochondrial pellet was resuspended in MSH buffer at a protein concentration of 80–100 mg/ml. The membrane was blocked for 1hr. Anti-Cytochrome C antibody (1:1000; Cell Signaling Technology, Beverly, MA, USA) and anti-COX IV antibody (1:1000; Cell Signaling Technology, Beverly, MA, USA) were diluted in TBST buffer (Tris-buffered saline/0.1% Tween 20) and incubated at 4°C overnight. The appropriate secondary antibody was used (1:10000 horseradish peroxidase anti-rabbit; Jackson ImmunoResearch Laboratories, West Grove, PA, USA) at room temperature in 1 hr. Visualization was performed by enhanced chemiluminescence (Visual Protein Biotechnology Crop, Taiwan). The protein bands on the destained gels were quantified with the Kodak 1D Image Analysis Version 3.5 software package (Eastman Kodak Company, Rochester, NY, USA).

**Measurement of leukocyte count in bronchoalveolar lavage fluid (BALF)**

All experiments were terminated after 120 min of closed extracorporeal perfusion, and the lungs were removed and wet weights were measured. The lungs were lavaged twice by instilling saline (2.5 ml/lavage) in the left lung. Lavage fluids were centrifuged at 1,500 g at room temperature for 10 min. The white cell count was determined as previously described [4].

**Myeloperoxidase assay**

The concentration of myeloperoxidase (MPO), an index of neutrophil sequestration in the lungs, was measured as previously described [2, 3].

**Measurement of H_2_O_2_ levels in BALF**

Samples were centrifuged 1000 × g within 30 minutes and the supernatants retained. Add 50μl of the H_2_O_2_ Reaction Mix (46μl Assay buffer, 2μl OxiRed^TM^ Probe solution and 2μl HRP solution; BioVision, USA) to each samples and H_2_O_2_ standards. Mix well. Incubate at room temperature for 10min. The absorbance was read at 570 nm (SpectraMax M5, Molecular Devices, USA).

**Protein carbonyl and thiobarbituric acid reactive substances (TBARS) assays in BALF**

ROS was assessed by protein carbonyl and TBARS assays that are indices of protein and lipid peroxidation and oxidative stress, respectively. Protein carbonyl content was measured by Protein Carbonyl Assays (**Geneteks Biosciences, Inc.,** Taiwan). TBARS level were measured using [OxiSelect™ TBARS Assay Kit](javascript:ShowContent('Description_Layer_284')) (**Geneteks Biosciences, Inc., Taiwan**). Protein carbonyl and TBARS of circulating perfusate, BALF, and lung tissue were measured, respectively. The carbonyl protein concentration of soluble protein fractions was determined [2]. Protein carbonyl content was measured by forming labeled protein hydrazone derivatives, using 2,4-dinitrophenylhydrazide. These derivatives were sequentially extracted with 10% (vol/vol) trichloroacetic acid, followed by treatment with 1:1 ethanol/ethylacetate (vol/vol), and re-extraction with 10% trichloroacetic acid. The resulting precipitate was dissolved in 6M urea hydrochloride. The spectrophotometric difference from a 2,4-dinitrophenylhydrazide protein blank was used to calculate the nmol of 2,4-dinitrophenylhydrazide incorporated per milligram of protein. The results are shown for each sample read at 370 nm. TBARS level in the serum was determined [2]. A 0.5ml volume of serum was acidified with 2.5ml of 1.22M trichloroacetic acid (TCA)/0.6M hydrochloric acid (HCl) and left to stand at room temperature for 15 minutes. Next, 1.5ml of 0.67% thiobarbituric acid (TBA)/0.05M sodium hydroxide (NaOH) was added. The samples were incubated in a 100°C water bath for 30 minutes. They were left to cool at room temperature before the addition of 4ml of n-butanol. After thorough mixing, the mixture was centrifuged for 10 minutes at 3000rpm. The absorbance was measured spectrophotometrically at 532 nm.

**Glutathione (GSH) assay in lung tissue**

In a separate group of animals the right lung was used for GSH assay. The lung was washed in PBS, pH 7.2, blotted in 10 mL of ice-cold 5% MPA solution, then thoroughly homogenized for 2 min and centrifuged at 10 000 *g* for 10 min at 4°C. The upper clear aqueous phase was collected and assayed within 4 h. The protein content of the lysate was measured using a detergent compatible protein assay (Bio-Rad Laboratories, Hercules, CA, USA). Total lung GSH was measured using a colorimetric Microplate Assay Kit (Oxford Biomedical Research, Oxford, MI, USA) based on the oxidation of GSH by DTNB. The GSH standards and treated samples were added to microtiter plate wells, followed by DTNB and glutathione reductase. Addition of NADPH initiated the progressive reduction of DTNB by GSH, causing an increase in absorbance that was monitored at 405 nm. The rate of change in absorbance over 4 min is proportional to the GSH concentration, which was reported as mg GSH/mg of protein.

**Cytokines and prostaglandin E2 assays**

The levels of interleukin-1β (IL-1β), tumor necrosis factor-α (TNF-α), macrophage inflammatory protein 2 (MIP-2) and interleukin-10 (IL-10) in BALF were measured using commercial ELISA kits (R&D Systems, Oxon, UK). The absorbance was read at 450 nm (SpectraMax M5, Molecular Devices, USA). The content of prostaglandin E2 in BALF was measured using commercial ELISA kits (Cayman Chemical Company, USA). The absorbance was read at 450 nm (SpectraMax M5, Molecular Devices, USA).

**Western blotting analysis in lung tissue**

Lung tissues were homogenized using lysis buffer containing protease inhibitor cocktail (Roche, USA) and phosphatase inhibitor cocktail (Roche, USA). The total protein concentration in the extract was determined with a bicinchoninic acid (BCA) protein assay (Pierce, Rockford, IL, USA). 80 μg protein was separated on 10% sodium dodecyl sulphate polyacrylamide gel, and electro-transferred onto PVDF membrane (Millipore, USA). The membrane was blocked with 5% non-fat dry milk in TBS containing: 0.1% Tween 20 (TBST) for 1 h. Antibodies against phospho-p44/42 MAPK (ERK1/2), phospho-SAPK/JNK, phospho-p38 MAPK, anti-p44/42 MAPK (ERK1/2), anti- SAPK/JNK, and anti-p38 MAP Kinase (1:1000; Cell Signaling Technology, Beverly, MA, USA) were used. Antibodies against GADPH (1:10000; Lab Frontier, Korea), JNK1 (1:1000; Santa Cruz Biotechnology, USA), Caspase-3 (1:2000; Cell Signaling Technology, Beverly, MA, USA), BcL-2 (1:1000; Cell Signaling Technology, Beverly, MA, USA), VCAM-1 (1:1000; abcam, UK) and ICAM-1 (1:1000; abcam, UK) were used. The appropriate secondary antibodies were used (1:10000 horseradish peroxidase anti-rabbit IgG; Jackson ImmunoResearch Laboratories, West Grove, PA, USA). Visualization was performed by enhanced chemiluminescence (Visual Protein Biotechnology Crop, Taiwan). The protein bands were quantified with the Kodak 1D Image Analysis (Eastman Kodak Company, Rochester, NY, USA).

**NF-**κ**B analysis of nuclear protein**

Lung tissue was homogenized with a Dounce tissue homogenizer in 5 ml solution A (0.6% Nonidet P-40, 150 mM NaCl, 10 mM HEPES, pH 7.9, 1 mM EDTA, 0.5 mM PMSF). The homogenates were centrifuged for 30 s at 2,000 rpm, and the supernatants were collected and centrifuged for 5 min at 5,000 rpm. The pelleted nuclei were resuspended at 4°C in 300 μl solution B (25% glycerol, 20 mM HEPES, pH 7.9, 420 mM NaCl, 1.2 mM MgCl_2_, 0.2 mM EDTA, 0.5 mM DTT, 0.5 mM PMSF, 2 mM benzamidine, 5 μg/ml pepstatin A, 5 μg/ml leupeptin, 5 μg/ml aprotinin) and incubated on ice for 20 min. Samples were centrifuged at 15,000 rpm for 1 min. The total protein concentration in the extract was determined with a bicinchoninic acid (BCA) protein assay (Pierce, Rockford, IL, USA). The membrane was blocked for 1hr. Anti-NF-κB antibody (1:1000; Cell Signaling Technology, Beverly, MA, USA) and anti-PCNA antibody (1:1000; Cell Signaling Technology, Beverly, MA, USA) were diluted in TBST buffer (Tris-buffered saline/0.1% Tween 20) and incubated at 4°C overnight. The appropriate secondary antibody was used (1:10000 horseradish peroxidase conjugated goat anti-rabbit IgG; Jackson ImmunoResearch Laboratories, West Grove, PA, USA) at room temperature in 1 hr. Visualization was performed by enhanced chemiluminescence (Visual Protein Biotechnology Crop, Taiwan). The protein bands on the destained gels were quantified with the Kodak 1D Image Analysis Version 3.5 software package (Eastman Kodak Company, Rochester, NY, USA). Anti-PCNA antibody was used as a loading control to correct the pixel values for NF-κB.

**TUNEL stain for apoptosis in lung tissue**

Lung slides coated with poly-L-lysine (Sigma, St. Louis, MO, USA) were deparaffinized and rehydrated using xylene and ethanol. The background was diminished by preincubating samples with 3% bovine serum albumin (BSA), 20% normal bovine serum in PBS, 30 min at RT. The specimens were then exposed 1 h at 37°C in a moist chamber to the labeling mix containing 0.135 U/mL calf TdT, 0.0044 nmol/mL digoxigenin-11-2’- deoxy-uridine-5’-triphosphate, and 1 mM Co chloride in distilled water. Following washing, the specimens were re-saturated in 3% BSA and 20% normal sheep serum, then treated (1 h at RT) with a 1.25 peroxidase U/mL dilution of peroxidase-labeled anti-digoxigenin sheep Fab fragment, followed by washing and 0.05% 3-3’-diaminobenzidine tetrahydrochloride (DAB) (Dako, USA) color reaction. Analysis was performed under Eclopse 80i microscope (Nikon, Japan) using Image Pro Plus 5.0 (Media Cybernetics, USA). The cells with positive TUNEL staining in nuclei was counted in 100 cells in 3 slides of immunohistochemical stain in each animal lung tissues. Two pathologists carried out the assessments of morphology blinded to the experimental condition.

**Immunohistochemistry of stem cell in lung tissue**

Lung slides coated with poly-L-lysine (Sigma, St. Louis, MO, USA) were deparaffinized and rehydrated using xylene and ethanol, and placed in 3% H_2_O_2_ for 15 min. The slides were incubated with a 1:100 dilution of monoclonal BrdU (Gene Tex, USA) and incubated at 4°C overnight, and stained with diaminobenzidine (DAB) (Dako, USA) and Mayer’s Hematoxylin (Dako, USA). Analysis was performed under Eclopse 80i microscope (Nikon, Japan) using Image Pro Plus 5.0 (Media Cybernetics, USA). Two pathologists carried out the assessments of morphology blinded to the experimental condition.

**Lung histopathology**

After the termination of each experiment, the lung tissue in right lower lobes were dissected and fixed immediately in 10% neutral buffered formalin. After fixation, the lung tissues were dehydrated through a graded series of alcohol, cleared in xylene, and embedded in paraffin. All sections were cut to 5μm and stained with hematoxylin/eosin (HE). The severity of perivascular, peribronchial, septal and alveolar edema as well as perivascular, interstitial and alveolar cell infiltration was examined by a scoring system. We developed a scoring method to measure the severity of acute lung injury, as follows: perivascular edema = 1; peribronchial edema=2; interstitial edema=2; alveolar edema=3; perivascular cell infiltration=2; interstitial cell infiltration=3; alveolar cell infiltration =4. A total of 20 scope views were examined for each lung tissue specimen. The sum of all the pathological scores was the score for each scope, and then we calculated the mean score of 20 scopes as the injury score for this lung tissue. Blind reviews were carried out by two pathologists, and the mean of these two scores was taken as the final score [4]. We developed a scoring method to measure the amount of pulmonary embolism, as follows. Pulmonary embolism was positive vessel obstruction by red cells and other cells. The total number of positive obstructive vessels was counted in 3 slides of hematoxylin/eosin stain in each animal lung tissues.

**REFERENCES**

1. [de Perrot M](http://www.ncbi.nlm.nih.gov/pubmed?term=de Perrot M%5BAuthor%5D&cauthor=true&cauthor_uid=12588712), [Liu M](http://www.ncbi.nlm.nih.gov/pubmed?term=Liu M%5BAuthor%5D&cauthor=true&cauthor_uid=12588712), [Waddell TK](http://www.ncbi.nlm.nih.gov/pubmed?term=Waddell TK%5BAuthor%5D&cauthor=true&cauthor_uid=12588712), [Keshavjee S](http://www.ncbi.nlm.nih.gov/pubmed?term=Keshavjee S%5BAuthor%5D&cauthor=true&cauthor_uid=12588712). Ischemia-reperfusion-induced lung injury. Am J Respir Crit Care Med. 2003;167:490-511.
2. Chiang CH, Chuang CH, Liu SL. Apocynin attenuates ischemia-reperfusion lung injury in an isolated rat lung model. Transl Res. 2011;158:17-29.
3. [Chiang CH](http://www.ncbi.nlm.nih.gov/pubmed?term=Chiang CH%5BAuthor%5D&cauthor=true&cauthor_uid=21618001), [Chuang CH](http://www.ncbi.nlm.nih.gov/pubmed?term=Chuang CH%5BAuthor%5D&cauthor=true&cauthor_uid=21618001), [Liu SL](http://www.ncbi.nlm.nih.gov/pubmed?term=Liu SL%5BAuthor%5D&cauthor=true&cauthor_uid=21618001), [Lee TS](http://www.ncbi.nlm.nih.gov/pubmed?term=Lee TS%5BAuthor%5D&cauthor=true&cauthor_uid=21618001), [Kou YR](http://www.ncbi.nlm.nih.gov/pubmed?term=Kou YR%5BAuthor%5D&cauthor=true&cauthor_uid=21618001), [Zhang H](http://www.ncbi.nlm.nih.gov/pubmed?term=Zhang H%5BAuthor%5D&cauthor=true&cauthor_uid=21618001). Apocynin attenuates ventilator-induced lung injury in an isolated and perfused rat lung model. Intensive Care Med. 2011;37:1360-1367.
4. [Chiang CH](http://www.ncbi.nlm.nih.gov/pubmed?term=Chiang CH%5BAuthor%5D&cauthor=true&cauthor_uid=9262456), [Hsu K](http://www.ncbi.nlm.nih.gov/pubmed?term=Hsu K%5BAuthor%5D&cauthor=true&cauthor_uid=9262456), [Yan HC](http://www.ncbi.nlm.nih.gov/pubmed?term=Yan HC%5BAuthor%5D&cauthor=true&cauthor_uid=9262456), [Harn HJ](http://www.ncbi.nlm.nih.gov/pubmed?term=Harn HJ%5BAuthor%5D&cauthor=true&cauthor_uid=9262456), [Chang DM](http://www.ncbi.nlm.nih.gov/pubmed?term=Chang DM%5BAuthor%5D&cauthor=true&cauthor_uid=9262456). PGE1, dexamethasone, U-74389G, or Bt2-cAMP as an additive to promote protection by UW solution in I/R injury. J Appl Physiol. 1997;83:583-590.
